# Facial Nerve Regeneration in Immunodeficient Rats Using a Bio 3D Conduit Fabricated From Human Dental Pulp Stem Cells

**DOI:** 10.1155/sci/1923945

**Published:** 2025-08-25

**Authors:** Yuri Matsui-Chujo, Ayano Hatori, Monika Nakano, Yuki Kanno, Ryosuke Ikeguchi, Tomoki Aoyama, Kazuaki Fujita, Yudai Miyazaki, Yoko Torii, Shizuka Akieda, Daichi Chikazu, Yoko Kawase-Koga

**Affiliations:** ^1^Department of Oral and Maxillofacial Surgery, Tokyo Medical University, 6-7-1 Nishishinjuku, Shinjuku-ku, Tokyo 160–0023, Japan; ^2^Department of Oral and Maxillofacial Surgery, Tokyo Women's Medical University, 8–1 Kawadacho, Shinjuku-ku, Tokyo 162–0054, Japan; ^3^Department of Endodontology, School of Dental Medicine, University of Connecticut Health University of Connecticut Health, Farmington, Connecticut 06030, USA; ^4^Department of Rehabilitation Medicine, Kyoto University Graduate School of Medicine, Shogoin Kawara-cho 54, Sakyo-ku, Kyoto 606–8507, Japan; ^5^Department of Orthopeaedic Surgery, Kyoto University Graduate School of Medicine, Shogoin Kawara-cho 54, Sakyo-ku, Kyoto 606–8507, Japan; ^6^Human Health Sciences, Graduate School of Medicine, Kyoto University, Shogoin Kawara-cho 54, Sakyo-ku, Kyoto 606–8507, Japan; ^7^Cyfuse Biomedical K.K., South Tower-1F., Sumitomo Fudosan, 3-5-27Mita, Minato-ku, Tokyo 108–6301, Japan

**Keywords:** bio 3D printer, dental pulp stem cells, facial nerve, nerve conduit, nerve regeneration, peripheral nerve

## Abstract

Tumor surgery or trauma in the maxillofacial region may cause injuries to peripheral nerves, such as facial nerves. The gold standard of treatment for peripheral nerve injury has been autologous nerve grafting. Since new peripheral nerve regeneration technologies are required, three-dimensional (3D) structures fabricated only from cells by using Bio 3D printers are attracting attention. Dental pulp stem cells (DPSCs) are a promising option as a cell source because of their high clonogenic, proliferative, and multidifferentiation potentials. In this study, nerve conduits were fabricated from DPSCs using a Bio 3D printer, and their potential for nerve regeneration was evaluated in a rat facial nerve injury model. DPSCs were obtained from wisdomteeth of patients and cultured. A 5 mm Bio 3D conduit was fabricated by using a Bio 3D printer. Six F344 rnu-/rnu- rats with immune deficiency (10 weeks old, body weight: 190–240 g) were divided into two groups: a Bio 3D group (*n* = 3) and a silicone tube group (*n* = 3). The 5 mm Bio 3D conduits and silicone tubes were transplanted into 4 mm defects. Evaluation was performed at 12 weeks after the surgery. The whiskers of immunodeficient rats in both groups were moving. The number of myelinated axons was larger in the Bio 3D group than in the silicone group. Myelinated axon diameter (MAD) and myelin thickness (MT) of regenerated axons in the Bio 3D group were significantly greater than those in the silicone group (MAD: *p*  < 0.01, MT: *p*  < 0.05). In this study, we confirmed the nerve regeneration potential of Bio 3D structures fabricated from DPSCs that were transplanted into a rat model of facial nerve injury.

## 1. Introduction

Facial nerve palsy in the maxillofacial region frequently occurs as a peripheral nerve injury that can be caused by fractures, cancers, and orthognathic surgery. After facial nerve injury, it is difficult for the facial muscles on the injured side to move, resulting in facial asymmetry. Thus, facial nerve paralysis not only causes dysfunction of speech and mastication but also causes esthetic problems [1]. Recently, autologous nerve grafting has been the gold standard treatment for nerve injuries [2–4]. However, it has several problems, including a limited supply of nerves, pain, and sensory disturbance at the donor site, and risk of neuroma formation [5]. As an alternative treatment, artificial nerves that do not require a donor nerve and mimic the nerve itself have been developed. However, artificial nerves are inferior to autologous nerve grafting because they do not have supporting cells, blood vessels, and growth factors and consist only of scaffolding materials [4]. There is also the risk of infection because artificial nerves are composed of absorbable artificial materials, such as polyglycolic acid, polylactic acid, and absorbable collagen [6, 7]. In previous studies, Bio 3D printers were used to fabricate spheroids from skin fibroblasts, bone marrow mesenchymal stem cells (BMSCs) and human induced pluripotent (iPS) stem cell-derived MSCs, and 3D nerve conduits made of cells were created by only passing the layers through thin needles according to predesigned 3D data [8–12]. This process is called the Kenzan method. Using a Bio 3D printer, it is possible to fabricate 3D structures of suitable length, thickness, and diameter for implantation. Yurie et al. [8, 10] established a method for transplanting a 3D nerve conduit in a rat sciatic nerve injury model and reported the effectiveness of sciatic nerve regeneration.

Dental pulp stem cells (DPSCs) are MSCs that have high proliferative ability, clonogenic, and multilineage differentiation potential, such as neurogenic, osteogenic, adipogenic, and so on [13, 14]. DPSCs can be easily harvested from unwanted teeth such, as orthodontic extractions or wisdom teeth. Furthermore, they are less invasive than other stem cells and do not raise ethical issues. DPSCs derived from the neural crest [15] have been reported to have the potential for differentiation of neurons and secretion of neurotrophic factors (NFs) for axonal regeneration [16, 17]. Therefore, DPSCs are attracting attention as a source of nerve regeneration.

In this study, nerve conduits were fabricated from DPSCs using a Bio 3D printer and the potential for nerve regeneration was evaluated in a rat facial nerve injury model with the aim of establishing a novel method for facial nerve regeneration therapy.

## 2. Materials and Methods

### 2.1. Isolation and Culture of DPSCs

The study was conducted after obtaining written consent from all patients and was approved by the Institutional Ethics Committee of Tokyo Women's Medical University (Approval No. 2022-0029, approved on July 22, 2022). Human DPSCs were isolated from the dental pulp of extracted wisdom teeth (third molar) obtained from healthy donors. The dental pulp was digested with a solution containing 3 mg/mL collagenase type I (Sigma–Aldrich) at 37°C for 45 min to obtain a single-cell suspension through a 70 µm cell strainer. The cells were seeded in T225 flasks at a density of 2 × 10^6^ cells and cultured in DMEM low glucose medium (DMEM; NacalaiTesque) containing FBS, cell growth supplement and antibiotics. Cells were passageduntil 70% confluence. All experiments were carried out with passages every 4–5 days up to passage eight.

### 2.2. Spheroids From DPSCs Fabricating of Bio 3 Dimensional (3D) Conduits

Cells from passage eight were used for the present study. Cells were detached and collected using trypsin for 3 min, centrifuged at 1000 rpm for 5 min, and resuspended in a minimal volume of fresh medium. Cells were seeded in low cell adhesion 96-well plates (SUMILON PrimeSurface, Sumitomo Bakelite, Tokyo, Japan) at a density of 1.5 × 10^4^ cells/well and incubated at 37°C in a humidified 5% CO_2_ incubator. The cells aggregated and formed spheroids.

### 2.3. Production of Bio 3D Conduits Using the Kenzan Method

As previously reported, 3D conduits were produced from spheroids using a Bio-3DPrinter (Regenova, Cyfuse, Tokyo, Japan). A 3D model of the tubular structure was predesigned by the Bio 3D printer.

**Figure 1:**
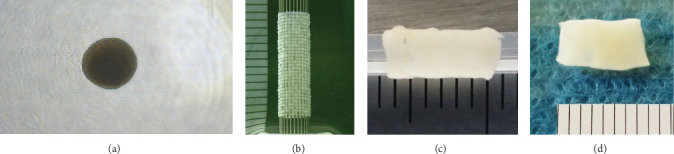
Process of fabricating the Bio 3D conduit. After building the tubular structure data using a Bio 3D printer, spheroids were laminated. (A) The spheroid. (B) Kenzan seen from the side. (C) Bio 3D conduit in a silicone tube before transplantation. (D) The Bio 3D conduit seen from the side.

The spheroids (Figure 1A) were aspirated from a 96-well plate into a thin suction nozzle and skewered into a circular needle array made of stainless steel to fabricate a 3D conduit using the Kenzan method (Figure 1B). It was circulated and cultured for approximately 1 week. The spheroids fuzed with each other and a single tubular shape was constructed within the Kenzan, after which the Kenzan was removed. The conduit was then transferred to a silicone tube and cultured in a perfusion bioreactor until the desired function and strength of the tissue were achieved to promote self-assembly of viable cells (Figure 1C). This process resulted in the fabrication of the Bio 3D conduit shown in Figure 1D, which was used for transplantation.

### 2.4. Animals

Animal experiments were conducted according to a protocol approved by the Animal Welfare and Management Committee of the Faculty of Medicine, Tokyo Medical University (Approval No. R5-128). Six immunocompetent adult F344 rnu-/rnu- male rats (10 weeks old, weighing 190–240 g, CLEA, Tokyo, Japan) were used for this study. They were divided into a Bio 3D group (*n* = 3) and a silicone group (*n* = 3). The Normal group was defined as the nerve on the contralateral side of the face that had not been transplanted with anything.

### 2.5. Transplantation

**Figure 2:**
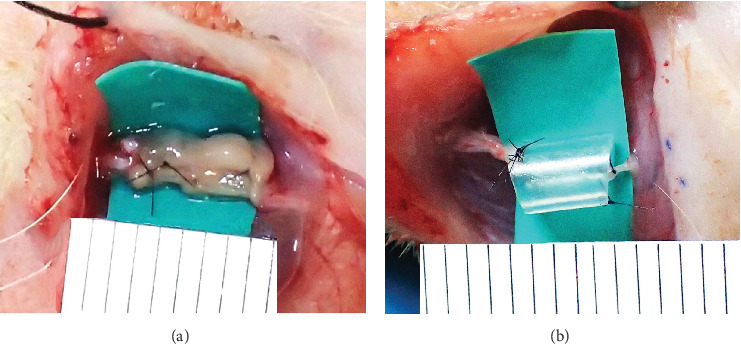
Photographs after grafting on nerve transection. (A) A 5 mm Bio 3D conduit was fabricated, and nerve cross-sections were sutured 0.5 mm into the conduit to form a 4 mm nerve gap. (B) The 5 mm silicone tube was sutured to the nerve cross-sections as in the Bio 3D group.

Each rat was anesthetized with medetomidine (0.375 mg/kg), midazolam (2.0 mg/kg), and butorphanol tartrate (2.5 mg/mL) via intraperitoneal injection and inhaled sevoflurane in oxygen for maintenance. The left facial skin was marked for incision. After disinfection, local anesthesia with Xylocaine with 80,000 ppm of epinephrine was performed. A facial skin incision was made with a No. 15 scalpel from the parotid gland to the lower border of the mandible. The buccal branch of the facial nerve was exposed and cut centrally with straight-tipped scissors to expose the cut, creating a nerve defect. A 5 mm Bio 3D conduit was inserted into this area and the proximal and distal nerve transects were fixed 0.5 mm into the tube, creating a 4 mm intertransverse gap in the conduit. Both proximal and distal nerve transects were fixed with 9–0 nylon suture. A silicone tube of 5 mm length and 1 mm in internal diameter was inserted using the same procedure (Figure 2). The wound was closed with skin sutures using 5–0 nylon sutures.

### 2.6. BASS (Black Light-Assisted Scoring System)

To assess functional recovery, beard movements were observed as reported by Miura et al. [18]. Briefly, rats were anesthetized by inhalation with 2% sevoflurane, after which they were restrained in a handmade plastic restraint suit. Black light paint (SO-KEN, Osaka, Japan) was applied to the whiskers and fixed-point photography was performed in a dark room at 30 cm above the rats' heads, while shining a black light (395 nm wavelength). Their whiskers were observed weekly and evaluated over a 12-week period.

### 2.7. Histological Analysis

**Figure 3:**
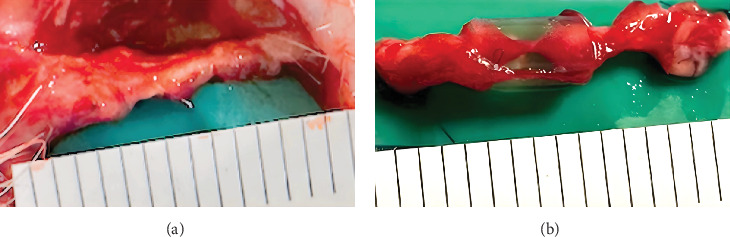
At 12 weeks after transplantation, the regenerated facial nerve was confirmed. (A) Bio 3D group. (B) Silicone group.

Twelve weeks after surgery, regenerated nerves were removed from rats in each group (Figure 3), fixed in 1% glutaraldehyde and 1.44% paraformaldehyde, postfixed in 1% osmine acid, and embedded in epoxy resin. Transverse sections (1 µm in thickness) were prepared from the central portion of the regenerated nerve. Sections were stained with 0.5% (w/v) toluidine blue solution and observed under an optical microscope (Olympus BX50, Tokyo, Japan) at 400× magnification to confirm regenerated nerves. Total myelinated axon counts were performed using an Image J software program (ver. 1.53, National Institutes of Health, Bethesda, MD, USA) as previously reported [19].

### 2.8. Morphological Analysis

Ultrathin crosssections (1 µm) from a sample of the epoxy resin block were stained with lead citrate and uranyl acetate and observed using a transmission electron microscope (TEM; model H-7000, Hitachi High-Technologies, Tokyo, Japan) at a magnification of 2000×. Image J was used to determine the minimum myelinated axon diameter (MAD, *a*) and bare axon diameter (*b*). From these two measurements, the myelin sheath thickness ([*ab*]/2) and G-ratio (*b*/*a*), the ratio of axon diameter to MAD, was calculated in each region. The averages of the four parameters were calculated for each field of view, and these results yielded an average value for all 11 fields of view for each sample of the two groups.

### 2.9. Statistical Analysis

Data are expressed as means ± standard deviation. All statistical analyses were performed by using Student's *t*-test in Microsoft Excel 2024 (Microsoft, Redmond, WA, USA) *p*  < 0.05 was considered significant.

## 3. Results and Discussion

### 3.1. Results

#### 3.1.1. Confirming the Strength of Bio 3D Conduit

At 48 h after seeding the DPSCs in low cell adhesion 96-well plates, the cells had aggregated and formed spheroids. The diameters of the spheroids were approximately 550 ± 25 µm. Using the Kenzan method, a Bio 3D conduit with an outer diameter of 3 mm and an inner diameter of 2 mm was fabricated. It had sufficient strength that it would not tear even when sutured with 9–0 nylon thread.

#### 3.1.2. Analysis of Beard Movement

Immediately after transection of the buccal branch of the left facial nerve, the movements of whiskers in the Bio 3D group and silicone group were worse than those in the healthy group. A difference between beard movements in the Bio 3D and silicone groups began to appear at 3 weeks postoperatively. At 12 weeks after the surgery, the left whiskers of rats in both groups were moving (Figure S1).

#### 3.1.3. Macroscopic Observation

At 12 weeks after transplantation, the same facial skin as before was incised to expose the nerve. It was confirmed that the nerve gap was filled in rats in both groups (Figure 3). Macroscopically, thicker nerves were regenerated in rats in the Bio 3D group. The regenerated nerves in the silicone group were thinner than those in the Bio 3D group.

#### 3.1.4. Histological and Morphometric Studies

**Figure 4:**
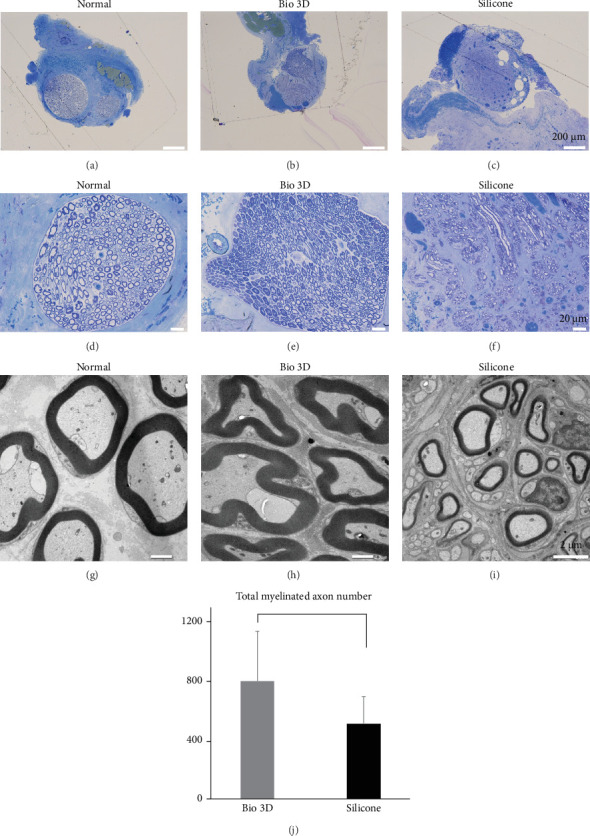
Histological evaluations of the regenerated facial nerve in the normal group, Bio 3D conduit group, and silicone tube group, and total number of myelinated axons in the regenerated nerves. Transverse sections shown for the Bio 3D and silicone groups were obtained from the distal region of the regenerated nerve. (A–F) Transverse sections stained with toluidine blue. Scale bars: 200 μm (A–C) and 20 μm (D–F). (G–I) Transverse section of transmission electron microscopy images (2000× magnification). Scale bars: 2 μm. (J) Total myelinated axon counts in the Bio 3D conduit group and silicone tube group.

**Figure 5:**
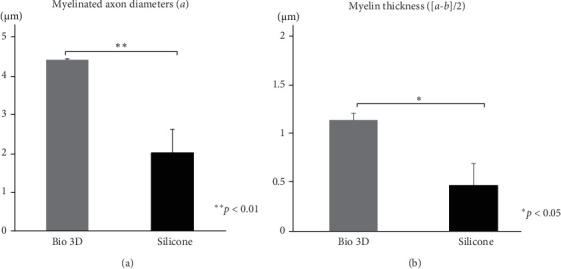
Morphological evaluation of myelinated axons of the regenerating facial nerve. (A) Myelinated axon diameters in the two groups. Brackets indicate significant differences (*⁣*^*∗∗*^*p*  < 0.01). (B) Myelin thicknesses in the two groups. Brackets indicate significant differences (*⁣*^*∗*^*p*  < 0.05).

After twelve weeks of transplantation, transverse sections of the distal region of the regenerated facial nerves were stained with toluidine blue. Numerous myelinated axons were observed in the Bio 3D group (Figure 4B,E). In contrast, the regenerated nerves in the silicone group exhibited signs of progressive degeneration and reduced axonal organization (Figure 4F). The total numbers of myelinated axons at 12 weeks after transplantation were 697 in the Bio 3D group and 508 in the silicone group (Figure 4J). There was no significant difference between the two groups (*p*=0.34). The diameter of myelinated axons was 4.343 ± 0.846 μm in the Bio 3D group, confirming regeneration of significantly thicker myelinated axons (*p*  < 0.01) than those in the silicone group (1.984 ± 0.033 μm) (Figure 5A). The mean myelin sheath thicknesses were 1.129 ± 0.319 μm in the Bio 3D group and 0.461 ± 0.096 μm in the silicone group, which was significantly greater in the Bio 3D group (*p*  < 0.05) (Figure 5B).

The mean G-ratios were 0.465 ± 0.051 for the Bio 3D group and 0.503 ± 0.115 in the silicone group. There was no significant difference between the two groups.

### 3.2. Discussion

The mainstream peripheral nerve regeneration therapy is autologous nerve grafting. However, autologous nerve transplantation has several disadvantages, as mentioned at the beginning of this article. On the other hand, artificial nerves made of polyglycolic acid and absorbable collagen have been developed, but the results for transplantation of artificial nerves are inferior to those for autologous nerve transplantation. The reason is that nerve regeneration is thought to require supporting cells, scaffolds, growth factors, and blood vessels [20]; however, artificial nerves have only scaffolds, and therefore are inferior to autologous nerve grafts. The Bio 3D printing technology focused on in this study has been used for regeneration of various tissues regeneration including skin, cartilage, blood vessels, and heart, as well as nerves.

In this study, a Bio 3D conduit derived from DPSCs was fabricated using a Bio 3D printer, and its efficacy in nerve regeneration was investigated. Its efficacy was evaluated in immunodeficient rats with facial nerve injury. This study is the first study showing thata Bio 3D conduit made of human DPSCs has significantly greater nerve regenerative potential than that of a silicone tube. A morphological study showed significant differences between the two groups in MAD and myelin thickness (MT) but not in the number of myelinated axons.

In previous studies, Bio 3D conduits were fabricated by dermal fibroblasts, bone marrow-derived stem cells and human-iPS stem cells [8–10]. The use of a Bio 3D conduit is expected to be a safe and effective method for promoting peripheral nerve regeneration because a Bio 3D conduit consists of only cells and does not have any scaffolding material. The effectiveness of a Bio 3D conduit for peripheral nerve regeneration was shown in those previous studies. In the process of nerve regeneration, a nerve undergoes axonal sprouting from the proximal end of the axon after injury if the cell body is healthy [21].

From a single axon, several regenerating axons will extend toward the distal end in bundles, and myelination begins as the newly formed Schwann cells encircle and invade the individual axons [22].

Also, as previously reported, nerve regeneration using Bio 3D conduits depends on the type of cells in the conduit [11]. There are several cell types that are suitable for nerve regeneration. Neural stem cells (NSCs) are stem cells that can differentiate into neurons or glial cells. Commercially available mouse C17.2 NSCs have shown a high rate of neuroblastoma formation in sciatic nerve injury rat models, but they were shown to be potentially unsuitable for treatment of the peripheral nervous system [23]. Although NSCs are found in multiple regions of the brain, harvesting them from the brain is a very invasive procedure [24].

MSCs are also thought to be an effective cell source for nerve regeneration. MSCs are isolated from adipose tissue, peripheral blood, amniotic fluid, umbilical cord, tendons and ligaments, hair follicles, synovium, olfactory mucosa, dental pulp, and fetal tissue [25], and they have multipotent differentiation potentials including fat, bone, muscle, and cartilage [26]. In the right circumstances, differentiation of MSCs into nonmesenchymal lineages, such as neurons, astrocytes, and Schwann-like cells can be induced to support nerve regeneration [27]. However, isolation of MSCs can be highly invasive for patients depending on the location of collection.

In 2000, Gronths et al. [14] reported that DPSCs have a significantly greater proliferative potential than that of BMSCs. DPSCs, like other MSCs and other stem cells, can multiply into nerve, fat, bone, and cartilage. In addition, DPSCs derived from the neural crest express neural markers even in the undifferentiated state [18]. It has also been reported that DPSCs can support nerve elongation by secreting NFs that can induce axon elongation and by differentiating into Schwann-like cells [28]. Thus, DPSCs are a promising cell source for nerve regeneration. For these reasons, nerve regeneration using Bio 3D conduits fabricated with DPSCs is an innovative treatment for easier and more efficient nerve regeneration.

There are several issues to be addressed in this study. First, there was no significant difference between the group in the movement of the whiskers. Rat whiskers are anatomically bilaterally innervated by the buccal and mandibular border branches of the facial nerve, and it may be difficult to evaluate their function due to the structure of the facial nerve in small animals [29]. Considering actual clinical practice, evaluation in medium and large animals is recommended for functional assessment. Second, it is necessary to evaluate how Bio 3D conduits derived from DPSCs affect regenerated nerves after transplantation. In future studies, it is necessary to transplant labeled Bio 3D conduits and evaluate whether DPSCs differentiate into neurons or induce regenerated axons as supporting cells. In this study, a 4 mm nerve gap was created, and a 5 mm Bio 3D conduit was fabricated.

In a previous study, a 12 mm Bio 3Dconduit fabricated by human dermal fibroblasts was transplanted to a 10 mm sciatic nerve defect in immunodeficient rats [30]. A Bio 3D conduit of 5 mm in outer diameter was fabricated in another study [19]. Therefore, we need to evaluate the efficacy for longer nerve gaps and large axial gaps in future studies with a view to clinical application.

Since it was possible to fabricate a Bio 3D conduit that was strong enough not to tear even with 9–0 nylon thread, we believe that it is possible to fabricate a long Bio 3D conduit that is strong enough for longer gaps and will be useful for clinical applications in the future.

## 4. Conclusions

In this study, a Bio 3D conduit derived from DPSCs was fabricated using a Bio 3D printer, and its efficacy in nerve regeneration was investigated. We evaluated the efficacy of the Bio 3D conduit in an immunodeficient rat model of facial nerve injury and confirmed significant nerve regeneration. Because the Bio 3D conduit is scaffold-free, the use of the Bio 3D conduit is expected to be a safe and efficient method for promoting peripheral nerve regeneration.

## Data Availability

The data presented during the current study are available upon reasonable request from the corresponding author.

## Supporting Information

Supporting Information Figure S1: Movements of the whiskers in the Bio 3D group and silicone group. The left side is the operated side.
