# Supplementary figures and images for: Facial Nerve Regeneration in Immunodeficient Rats Using a Bio 3D Conduit Fabricated From Human Dental Pulp Stem Cells
Source: Stem Cells Int. 2025 Aug 25;2025:1923945. doi: 10.1155/sci/1923945 (PMC12401602; doi:10.1155/sci/1923945)

## Slide 1
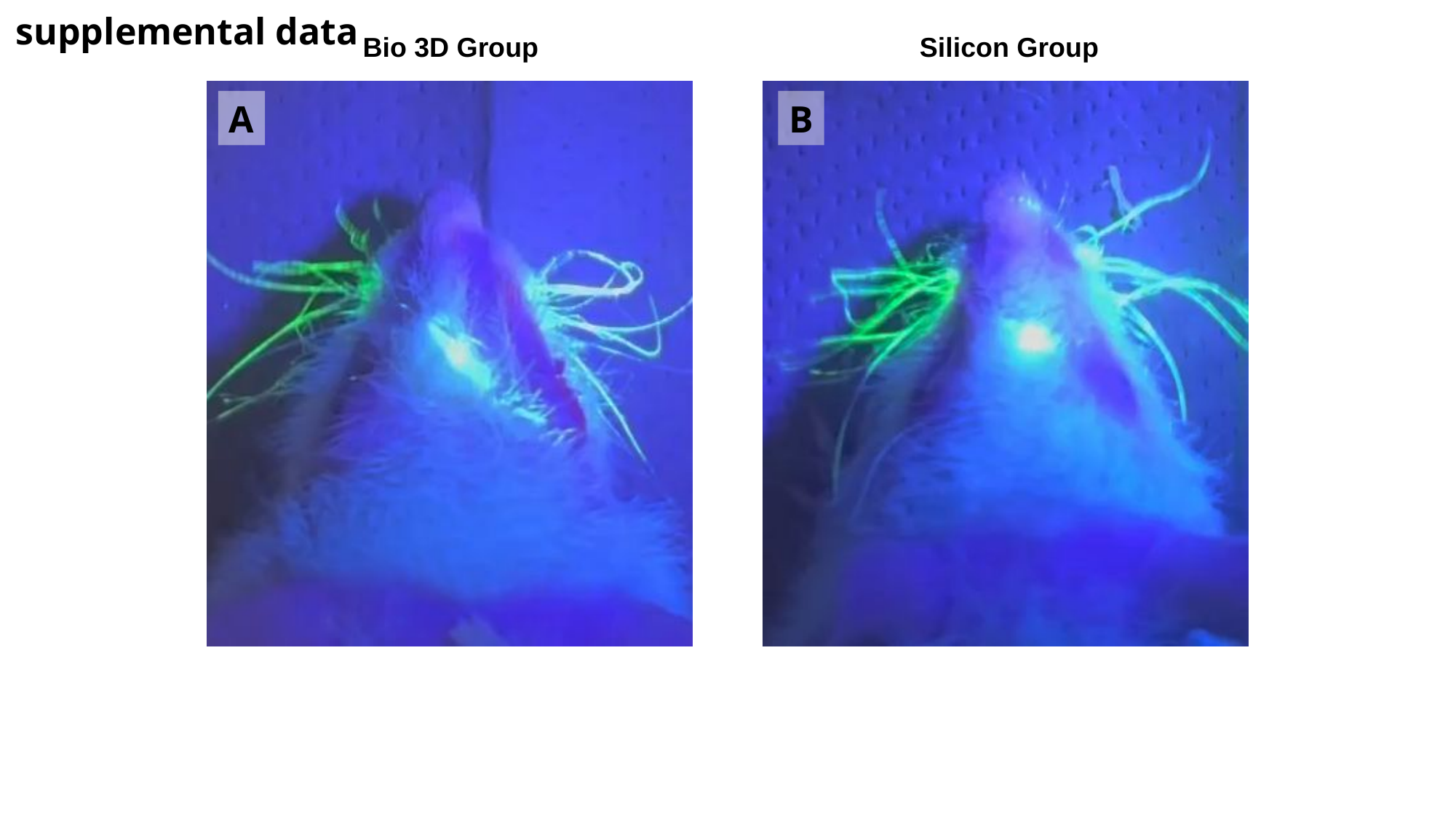

supplemental data
Bio 3D Group
Silicon Group
B
A

Supplement: Supporting Information — Figure S1: Movements of the whiskers in the Bio 3D group and silicone group. The left side is the operated side. [file 1923945.f1.pptx]
